# Microstructure and Texture Evolution in Cold-Rolled and Annealed Oxygen-Free Copper Sheets

**DOI:** 10.3390/ma17102202

**Published:** 2024-05-08

**Authors:** Jing Qin, Xun Li, Dongsheng Wang, Chen Zhou, Tongsheng Hu, Jingwen Wang, Youwen Yang, Yujun Hu

**Affiliations:** 1 School of Mechanical Engineering, Tongling University, Tongling 244000, China; 2 Key Laboratory of Construction Hydraulic Robots of Anhui Higher Education Institutes, Tongling University, Tongling 244000, China; 3 New Copper-based Material Industry Generic Technology Research Center of Anhui Province, Tongling 244000, China; 4 Anhui Joint Key Laboratory of Critical Technologies for High-End Copper-Based New Materials, Tongling 244000, China; leexun1225@163.com (X.L.); zc200610@126.com (C.Z.); huts@tlys.cn (T.H.); 5 Jinvi Copper Corporation, Tongling Nonferrous Metals Group Holding Co., Ltd., Tongling 244000, China; 6 School of Mechanical and Electrical Engineering, Jiangxi University of Science and Technology, Ganzhou 341000, China; yangyouwen@jxust.edu.cn; 7 School of Materials Science and Engineering, Jiangxi University of Science and Technology, Ganzhou 341000, China; hyj0525@126.com

**Keywords:** microstructure, texture, copper sheet, rolling reduction, annealing twin

## Abstract

Commercial oxygen-free copper sheets were cold-rolled with reduction rates ranging from 20% to 87% and annealed at 400, 500 and 600 °C. The microstructure and texture evolution during the cold-rolling and annealing processes were studied using optical microscopy (OM), scanning electron microscopy (SEM) and electron back-scattered diffraction (EBSD). The results show that the deformation textures of {123}<634> (S), {112}<111> (Copper) and {110}<112> (Brass) were continuously enhanced with the increase in cold-rolling reduction. The orientations along the α-oriented fiber converged towards Brass, and the orientation density of β fiber obviously increased when the rolling reduction exceeded 60%. The recrystallization texture was significantly affected by the cold-rolling reduction. After 60% cold-rolling reduction, Copper and S texture components gradually decreased, and the {011}<511> recrystallization texture component formed with the increase in annealing temperature. After 87% cold-rolling reduction, a strong Cube texture formed, and other textures were inhibited with the increase in annealing temperature. The strong Brass and S deformation texture was conducive to the formation of a strong Cube annealing texture. The density of the annealing twin boundary decreased with the increase in annealing temperature, and more annealing twin boundaries formed in the oxygen-free copper sheets with the increase in cold-rolling reduction.

## 1. Introduction

The rapid advancement of new energy vehicles, aerospace, high-speed railways, lead frames and home appliances has raised higher demands for the overall performance of copper sheets and strips. They are required to possess not only high strength and electrical and thermal conductivity but also excellent stamping, bending, drawing and other forming capabilities [1,2,3]. The formability is generally influenced by the microstructure and crystallographic texture. For instance, a strong {001}<100> (Cube) texture is unfavorable for deep drawing due to significant r-value differences between the 0° and 45° directions, whereas the coexistence of {123}<634> (S), {112}<111> (Copper) and {110}<112> (Brass) and {110}<001> (Goss) texture components contributes to improving the average r-values for deep drawability [4]. The texture control was found to be highly effective in achieving superior formability, with the fundamental principle being the mastery of the deformation and recrystallization evolution law for copper sheets.

Previous research has demonstrated that the texture evolution of copper and copper alloys was influenced by factors such as chemical composition, grain size, second-phase precipitation, deformation amount, deformation mode, deformation and annealing temperatures [5,6,7]. In high-stacking-fault-energy (SFE) metals like pure copper, the Copper texture component became stronger after rolling, whereas in low-SFE alloys like brass, the formation of the Brass texture was promoted due to twin rotation [8]. The addition of alloy elements affected the change in SFE and the precipitation of the second phases, thus impacting texture formation and development. Additionally, the initial grain size also affected the type of deformation texture. For instance, after 97% rolling reduction, a typical Copper texture was obtained in the sample with coarse grains (24 μm), while a Brass texture was obtained for the sample with ultrafine grains (0.36 μm) [9]. Furthermore, in diffusion-precipitated reinforced copper alloys, texture evolution was correlated with the size, distribution and volume fraction of the second-phase particles [10,11]. With the increase in aging temperature, the secondary phase precipitation of the Cu-Ni-Si alloy after aging treatment gradually decreased the Copper and Goss texture components, while the Cube texture component gradually increased [12]. The deformation mode was also a key factor influencing the type and distribution of textures. Copper and Brass texture components remained stable during deformation for face-centered cubic (FCC) metals with the dislocation slip as the primary deformation mechanism. With an increase in twinning, the Copper texture gradually weakened, while the Brass texture strengthened, and shear deformation enhanced Brass texture. The rolling reduction rate was also a crucial process parameter for controlling the deformation microstructure, with some deformation textures being enhanced with increasing degrees of deformation. In the case of the Cu-Ni-Si alloy, Cu-Cr-Zr alloy and α brass, as cold-rolling reduction rates increased, the Copper texture gradually transitioned to Brass texture [13]. Different rolling methods also led to changes in deformation textures, and asymmetric rolling caused the grain orientation to rotate around the transverse direction of the sample [14]. 

Heat treatment following deformation can weaken the deformation texture and lead to the formation of the recrystallization texture. The great influence on the grain orientation is the grain boundary migration, and the difference and distribution of grain orientation determine the formation and development of the recrystallization texture [15]. The S deformation texture has been frequently associated with the Cube recrystallization texture in FCC metals, exhibiting a ~40° <111> rotational relation. Some findings suggest that Cube nuclei are more likely to grow into copper-oriented grains rather than S-oriented grains, indicating that Copper deformation texture plays a greater role in Cube recrystallization texture [16]. However, beyond Cube texture, the relationship between other types of recrystallization textures and deformation textures remains unclear and requires further investigation.

In this study, the microstructure and texture of oxygen-free copper sheets with different cold-rolling reduction rates and annealing temperatures were analyzed to elucidate the evolution of the microstructure and textures during rolling and annealing. The findings can serve as valuable references for texture control and process optimization in production.

## 2. Experimental Section

The experimental materials were 2.3 mm thick hot-rolled oxygen-free copper sheets (C10200, with a Cu content of 99.995 wt%). The chemical composition was determined by an optical emission spectrometer and is presented in Table 1. 

The hot-rolled copper sheets were subjected to cold-rolling on a four-high rolling mill with reduction rates of 20%, 40%, 60%, 80% and 87%, respectively. The work roll diameter of the rolling mill was 90 mm, the support roll diameter was 280 mm and the roll body length was 350 mm. Subsequently, the cold-rolled sheets were annealed at temperatures of 400, 500 and 600 °C for 5 min. Upon reaching the specified time, all specimens were rapidly cooled in ambient air. 

To observe the metallographic microstructure, each sample was ground, polished and etched by an etchant solution composed of 3 g FeCl_3_, 2 mL HCl and 96 mL H_2_O. The etched specimens were observed using a Motic BA310MET optical microscope (OM). The crystallographic texture of the samples was analyzed using a Zeiss ΣIGMA scanning electron microscope (SEM) and an Oxford instruments HKL-Chanel 5 electron back-scattered diffractometer (EBSD). Samples for EBSD testing were prepared by electropolishing at room temperature, using an electrolyte solution consisting of 825 volume phosphoric acid and 175 volume distilled water. 

## 3. Results and Discussion

### 3.1. Microstructure and Texture of Rolled Copper Sheets

**Figure 1:**
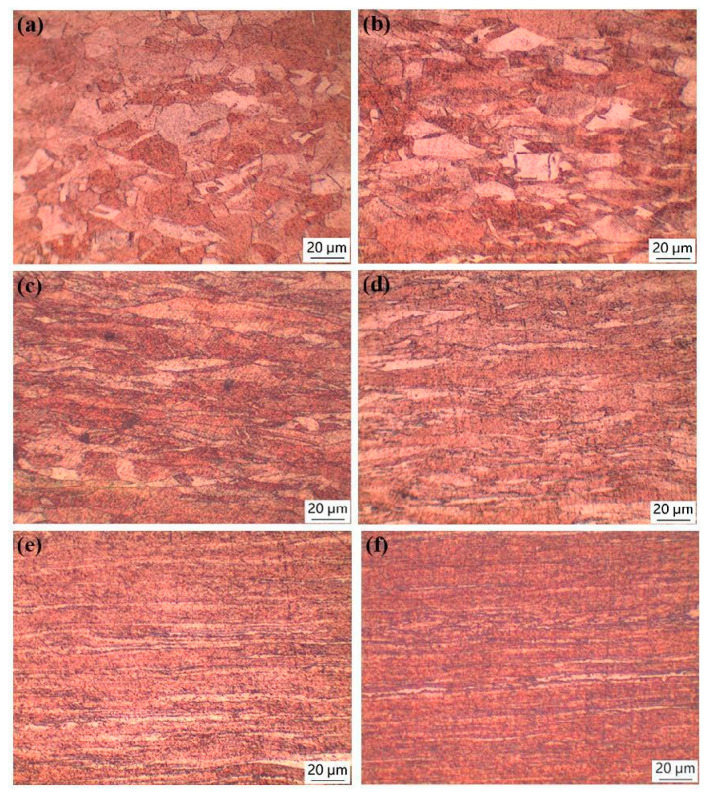
Metallographic microstructure of copper sheets under different rolling reductions: (**a**) 0; (**b**) 20%; (**c**) 40%; (**d**) 60%; (**e**) 80% and (**f**) 87%.

Figure 1 shows the metallographic microstructure of copper sheets with different rolling reduction rates. Initially, the grains were equiaxial before cold-rolling. However, as the cold-rolling reduction increased, the grains became elongated and flattened. When the reduction rate reached 80%, a distinct fibrous microstructure emerged.

**Figure 2:**
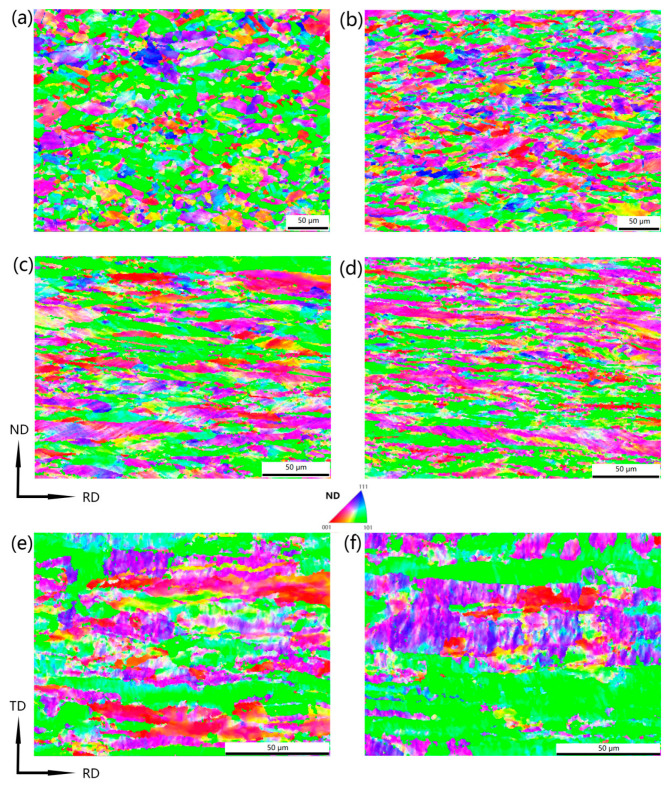
IPF maps of copper sheets under different rolling reductions: (**a**) 0; (**b**) 20%; (**c**) 40%; (**d**) 60%; (**e**) 80% and (**f**) 87%.

Figure 2 illustrates the inverse pole figure (IPF) maps of copper sheets subjected to varying cold-rolling reduction rates. As the reduction rate increased, dislocation slips resulted in grain orientation rotation and the formation of deformation bands. This led to a weakening and eventual disappearance of recrystallization textures, while simultaneously strengthening deformation textures.

**Figure 3:**
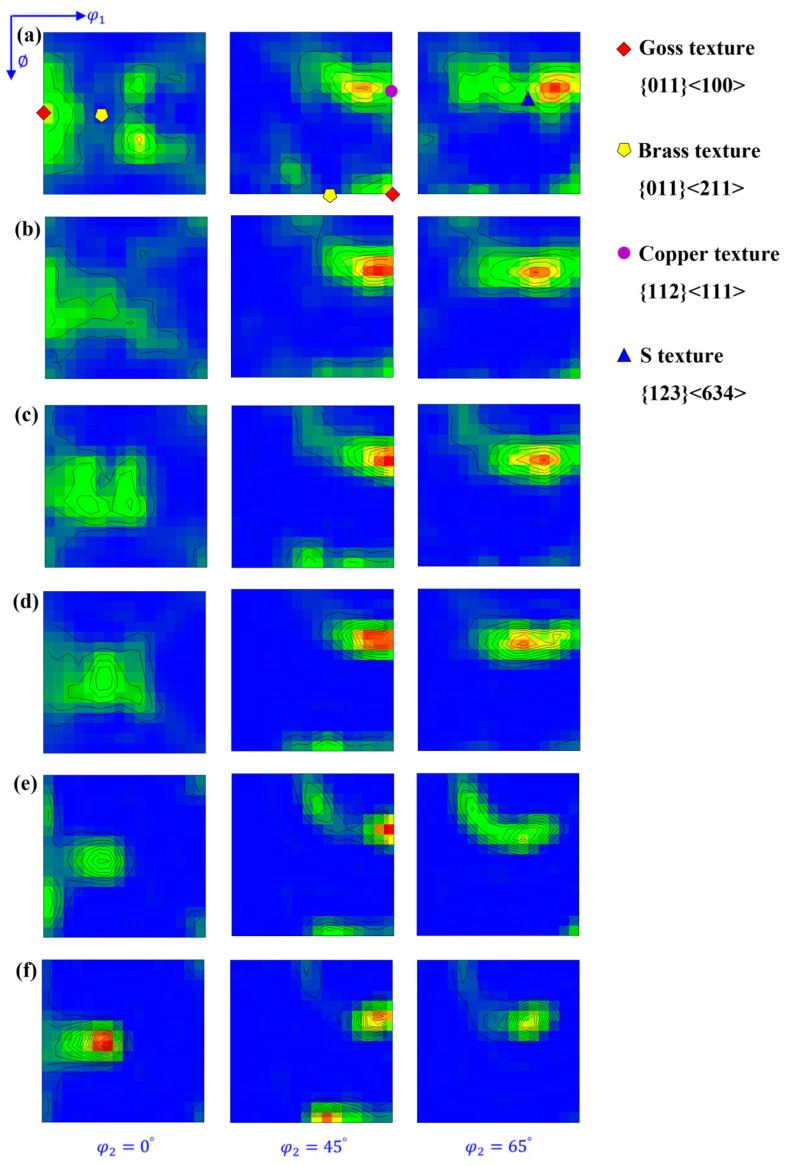
ODF maps at φ_2_ = 0°, 45° and 65° (levels: 1, 2, 3, 4 …) under different rolling reductions: (**a**) 0; (**b**) 20%; (**c**) 40%; (**d**) 60%; (**e**) 80% and (**f**) 87%.

Figure 3 shows the orientation distribution function (ODF) maps of copper sheets subjected to varying degrees of cold-rolling reduction. As the reduction rate increased, the Goss texture weakened, while the Copper, Brass and S textures consistently strengthened. In particular, there was a more pronounced concentration of S orientation.

**Figure 4:**
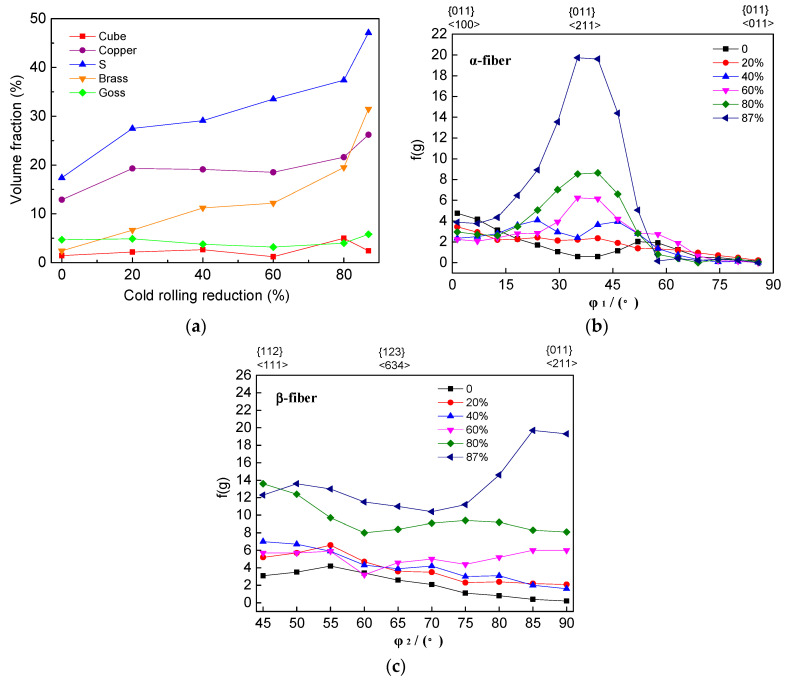
Volume fractions of main deformation texture components (**a**), variation curves of orientation densities along α-fiber (**b**) and β-fiber (**c**) under different rolling reductions.

Figure 4 illustrates the volume fractions of the main deformation texture components and the variation curves of orientation densities along α-fiber and β-fiber under different rolling reductions. As an FCC metal undergoing the rolling process, the grain orientation constantly changed and gradually converged towards stable lines, namely the α and β fibers. The predominant texture components on the α fiber included Goss and Brass, while Copper and S texture components were present on the β fiber. The position of Brass orientation in Euler space corresponds to the intersection of the α and β fibers [17]. As the cold-rolling reduction increased, the volume fractions of Copper, S and Brass texture components increased, and the orientation density of both α and β fibers increased. Beyond a 40% rolling reduction rate, there was a noticeable increase in {011}<211> texture component density, while {011}<100> remained relatively unchanged. Upon exceeding a 60% rolling reduction rate, there was a significant rise in the orientation density of the β fiber. Finally, when surpassing an 80% rolling reduction rate, there was maximal augmentation observed in {011}<211> texture component density. 

### 3.2. Microstructure and Texture of Annealed Copper Sheets

**Figure 5:**
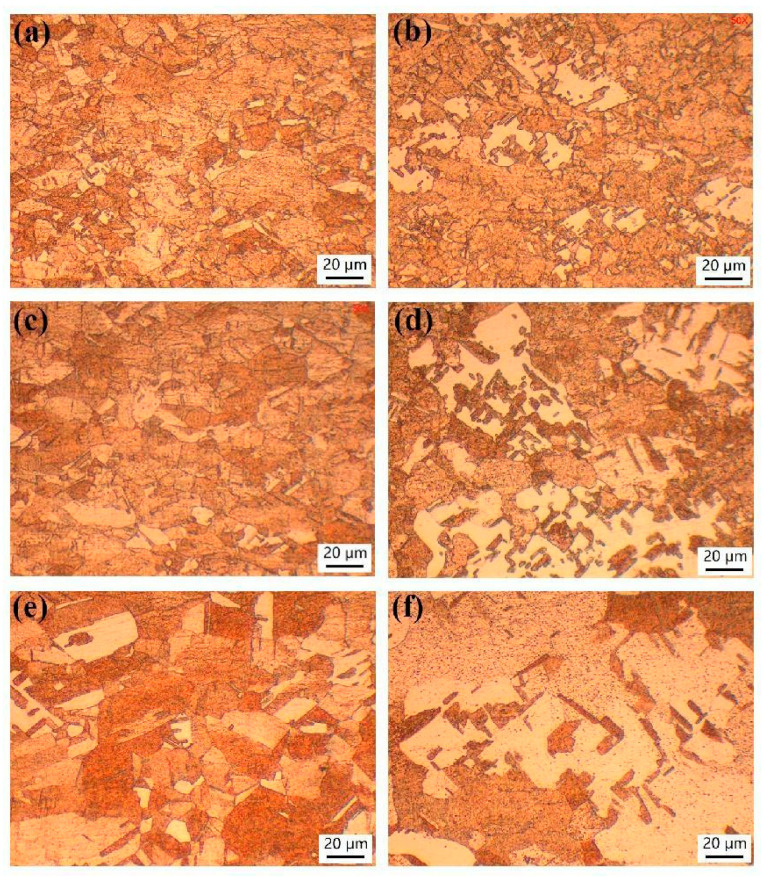
Metallographic microstructure of copper sheets with different rolling reduction rates and annealing temperatures: (**a**) 60%, 400 °C; (**b**) 87%, 400 °C; (**c**) 60%, 500 °C; (**d**) 87%, 500 °C; (**e**) 60%, 600 °C and (**f**) 87%, 600 °C.

Figure 5 shows the microstructure of copper sheets subjected to rolling reduction rates of 60% and 87%, followed by annealing at temperatures of 400, 500 and 600 °C. At the same annealing temperature, the average grain size of the sample with an 87% rolling reduction was larger than that of the sample with a 60% rolling reduction. Additionally, abnormal grain growth was observed in the sample with an 87% rolling reduction. 

**Figure 6:**
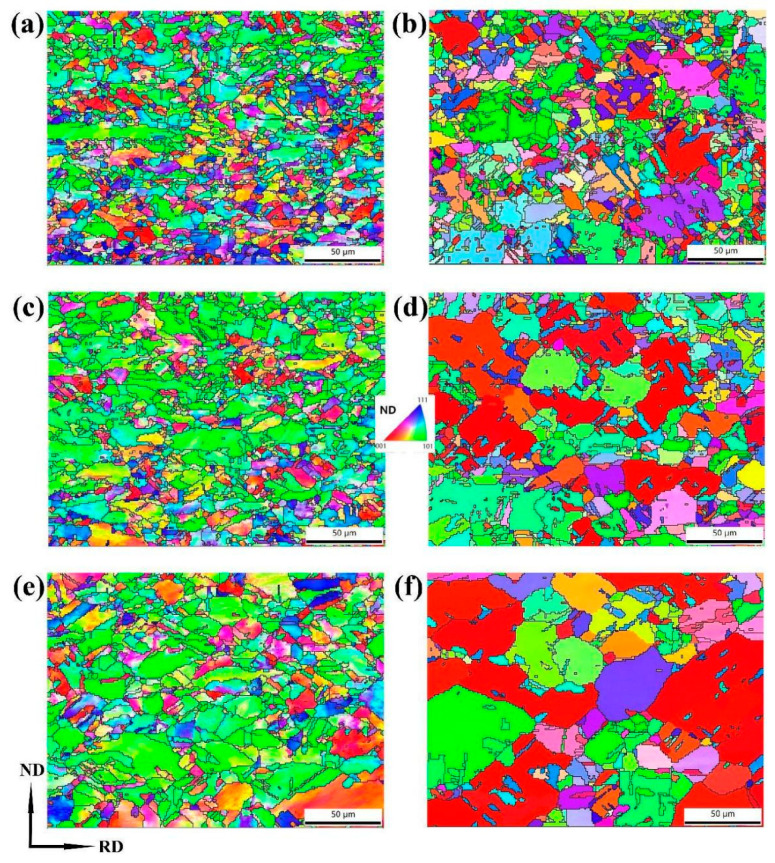
IPF maps of copper sheets with different rolling reduction rates and annealing temperatures: (**a**) 60%, 400 °C; (**b**) 87%, 400 °C; (**c**) 60%, 500 °C; (**d**) 87%, 500 °C; (**e**) 60%, 600 °C and (**f**) 87%, 600 °C.

Figure 6 shows the IPF maps of the annealed copper sheets with rolling reduction rates of 60% and 87%. After annealing at temperatures of 400, 500 and 600 °C, EBSD statistical analysis revealed that the average grain sizes for the sample with a 60% rolling reduction were measured at 8.4 μm, 9.4 μm and 10.7 μm, respectively; while for the sample with an 87% rolling reduction, they were recorded as being 8.8 μm, 10.5 μm and 14 μm, respectively. With increasing annealing temperature, {110} texture was observed to be enhanced in the sample with a 60% rolling reduction, whereas {100} texture was significantly strengthened in the sample with an 87% rolling reduction, and grains with the same orientation merged.

**Figure 7:**
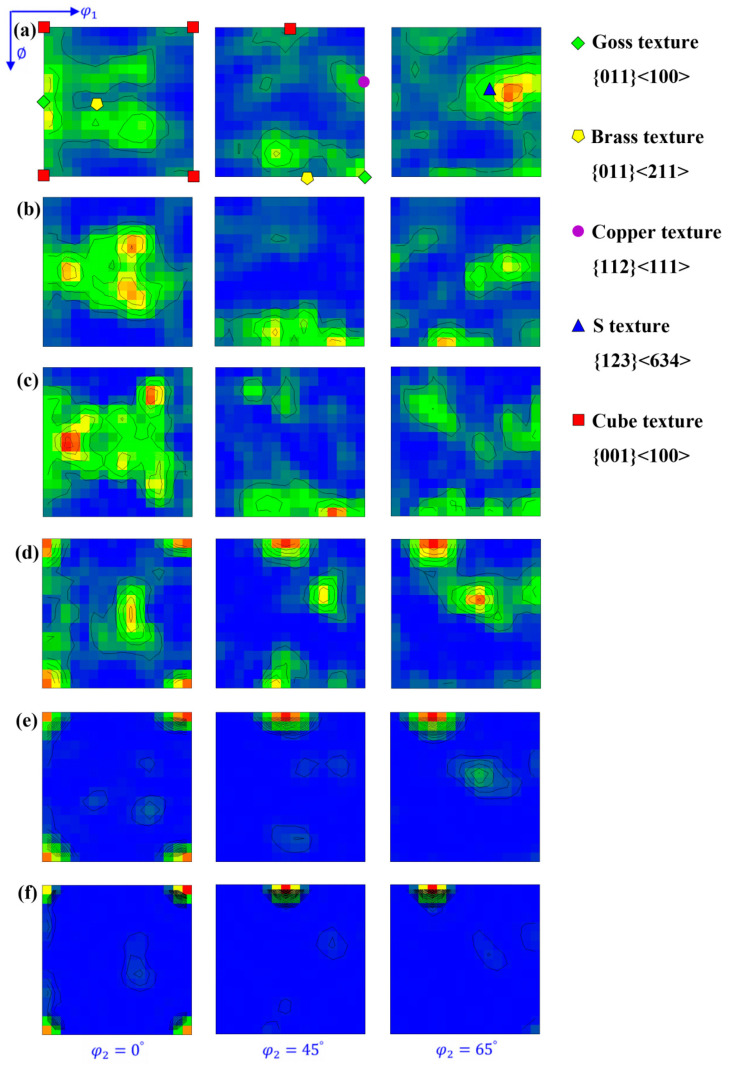
ODF maps at φ_2_ = 0°, 45° and 65° (levels: 1, 2, 3, 4 …) of copper sheets with different rolling reduction rates and annealing temperatures: (**a**) 60%, 400°C; (**b**) 60%, 500 °C; (**c**) 60%, 600 °C; (**d**) 87%, 400 °C; (**e**) 87%, 500 °C and (**f**) 87%, 600 °C.

Figure 7 shows the ODF maps of the annealed copper sheets with rolling reduction rates of 60% and 87%. In the copper sheets with a 60% rolling reduction, an increase in annealing temperature led to a gradual decrease in the deformation texture components of {112}<111> and {123}<634>, while the {011}<511> recrystallization texture component appeared and increased, which deviated 19° from Brass orientation. For copper sheets with an 87% rolling reduction, increasing annealing temperature resulted in decreased deformation texture components of {123}<634> and {011}<111>, while the recrystallization texture component of {001}<100> was consistently enhanced. Ultimately, the Cube texture emerged as the strongest and dominant one in the sample.

For copper sheets subjected to a 60% rolling reduction, the sample exhibited lower deformation energy storage, a lower recrystallization driving force and an extended incubation time before recrystallization compared to the sample with an 87% rolling reduction. Consequently, the sample with 60% rolling reduction displays a higher proportion of an unrecrystallized microstructure under the same annealing conditions. During the annealing process, the grain size gradually increased due to low-angle grain boundary migration and subgrain growth by rotation and coalescence. The orientation gradients as shown in Figure 6 suggest the rotation of grain orientation, resulting in the weakening of the {112}<111> and {123}<634> deformation textures and the strengthening of the {011}<511> texture component. 

**Figure 8:**
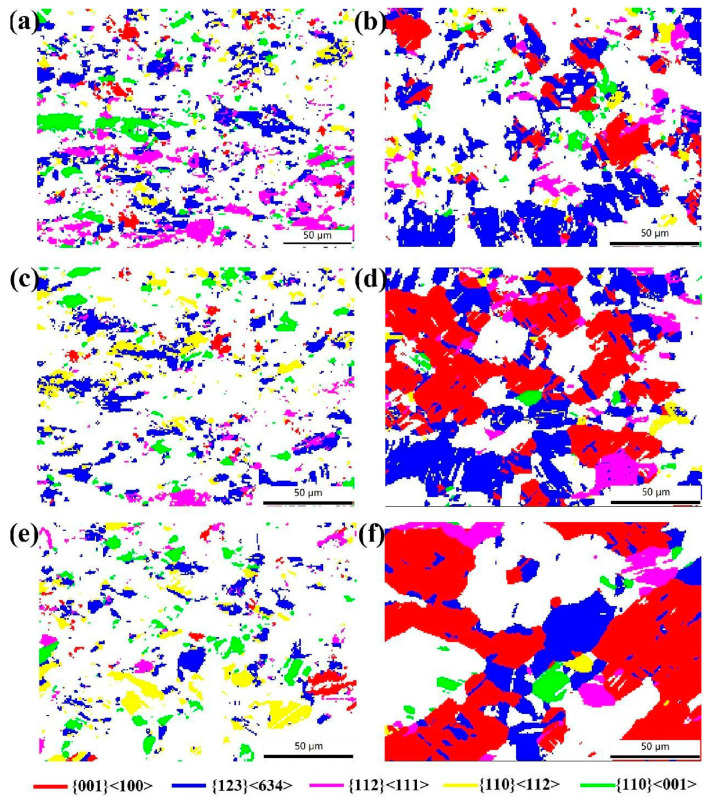
Maps of main texture components in copper sheets with different rolling reduction rates and annealing temperatures: (**a**) 60%, 400 °C; (**b**) 87%, 400 °C; (**c**) 60%, 500 °C; (**d**) 87%, 500 °C; (**e**) 60%, 600 °C and (**f**) 87%, 600 °C.

Figure 8 shows the distribution of the main texture components in the annealed copper sheets with rolling reduction rates of 60% and 87%. In the samples with a 60% rolling reduction, S-, Goss-, Copper- and Brass-oriented grains were present, showing a relatively uniform distribution. Cube-oriented grains were found to be the least abundant. Conversely, in the sample with an 87% rolling reduction, an increase in annealing temperature resulted in significantly larger Cube-oriented grains compared to other oriented grains. Additionally, there was a decrease in adjacent S-oriented grains which were predominant before annealing.

**Figure 9:**
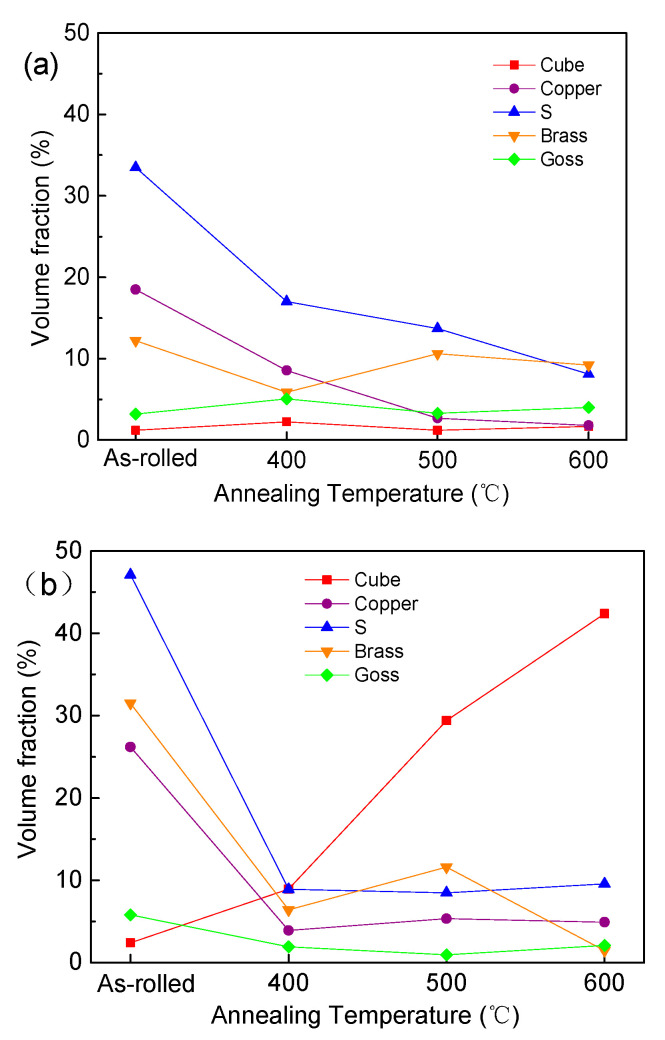
Volume fractions of main texture components in annealed copper sheets with different rolling reduction rates: (**a**) 60% and (**b**) 87%.

Figure 9 illustrates the volume fractions of the main texture components in annealed copper sheets subjected to rolling reduction rates of 60% and 87%. For samples with a 60% reduction, the volume fractions of the deformation texture components of S and Copper gradually decreased with increasing annealing temperature. The volume fraction of Brass texture components initially decreased and then increased, while Goss and Cube texture components remained at lower levels. In samples with an 87% reduction, the volume fractions of deformation texture components S, Brass and Copper significantly decreased at 400 °C, while the volume fraction of the Cube recrystallization texture component greatly increased with higher annealing temperatures. During the grain growth stage after recrystallization, the growth rate of Cube-oriented grains was notably higher than that of other oriented grains due to a selective growth advantage. Ultimately, Cube-oriented grains swallowed other oriented grains of smaller sizes due to the size effects, establishing the Cube texture as the predominant recrystallization texture.

**Figure 10:**
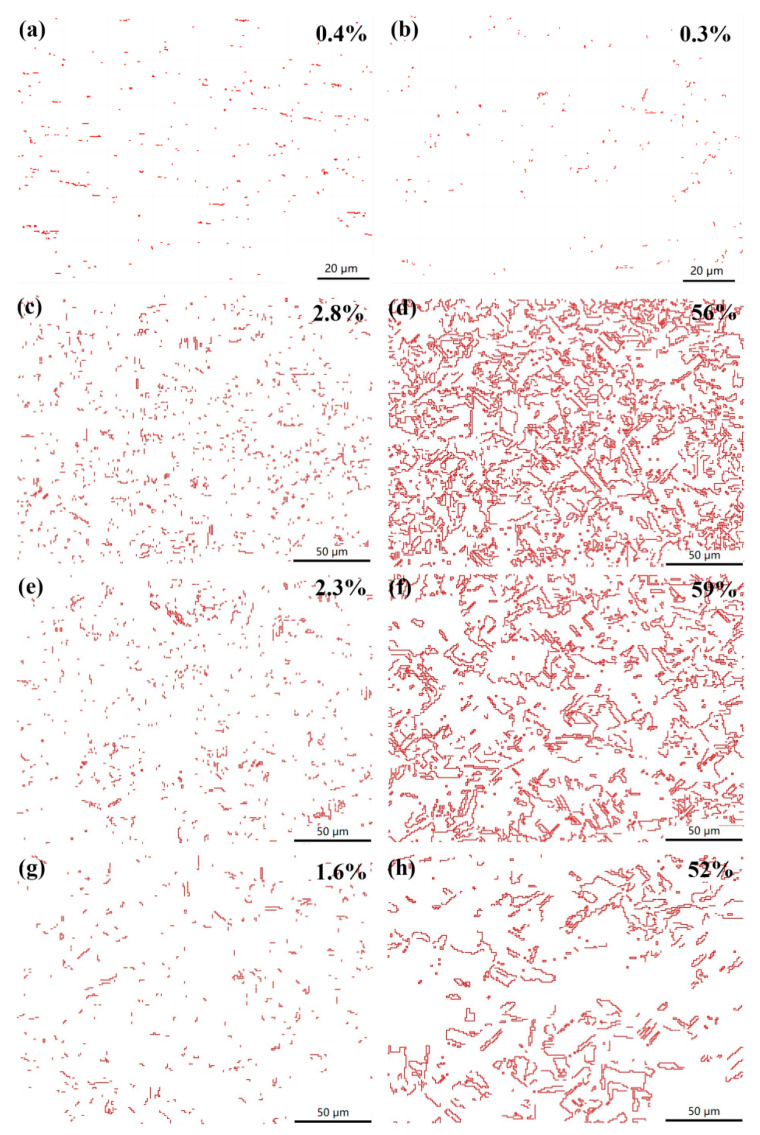
Distribution and density of twins in copper sheets with different rolling reduction rates and annealing temperatures: (**a**) 60%, As-rolled; (**b**) 87%, As-rolled; (**c**) 60%, 400 °C; (**d**) 87%, 400 °C; (**e**) 60%, 500 °C; (**f**) 87%, 500 °C; (**g**) 60%, 600 °C and (**h**) 87%, 600 °C.

Figure 10 shows the distribution and density of twins in copper sheets with rolling reduction rates of 60% and 87%. The statistical results obtained from EBSD indicate that the density of the 60° <111> (Σ3) twin was very low in both of the cold-rolled samples, with most of the twins forming during annealing. It is well known that annealing twins were prevalent in the recrystallized grains of pure copper with a high SFE [18,19]. Annealing twins form as a result of growth accidents on differently inclined {111} facets present on a migrating grain boundary, and growth twins also form due to growth accidents on the {111} planes [20]. Following annealing at temperatures of 400, 500 and 600 °C, the proportions of twin boundaries in the sample with a rolling reduction rate of 60% were measured at 2.8%, 2.3% and 1.6%, respectively, whereas those in the sample with an 87% rolling reduction rate were recorded at levels of 56%, 59% and 52%. These findings suggest that the density of annealing twins decreases with increasing annealing temperature, and the annealing twin boundaries increased by improving the cold-rolling reduction before recrystallization.

**Figure 11:**
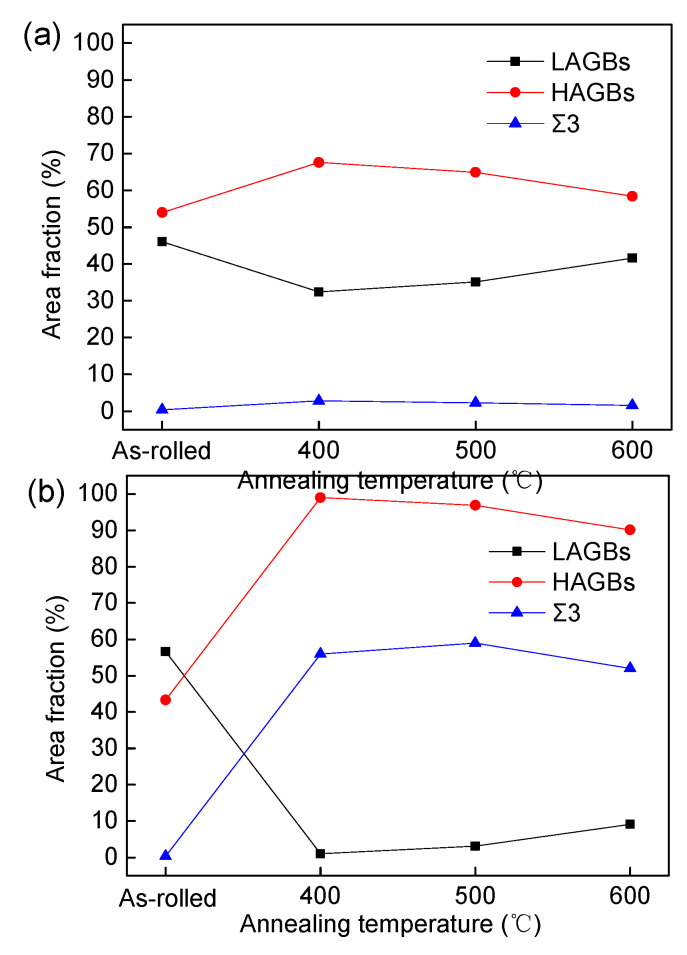
Grain boundary characteristics of copper sheets with different rolling reduction rates: (**a**) 60% and (**b**) 87%.

Figure 11 illustrates the area fractions for low-angle grain boundaries (LAGBs, with a misorientation angle *θ* < 15°), high-angle grain boundaries (HAGBs, with a misorientation angle *θ* ≧ 15°) and Σ3 twin boundaries in copper sheets subjected to rolling reductions of 60% and 87%. It is noteworthy that the presence of annealing twin boundaries significantly increased at 400 °C in the samples with an 87% rolling reduction, exhibiting a similar trend to HAGBs during the annealing process. In contrast, the area fraction of annealing twin boundaries remained consistently low in the samples with a 60% rolling reduction. 

**Figure 12:**
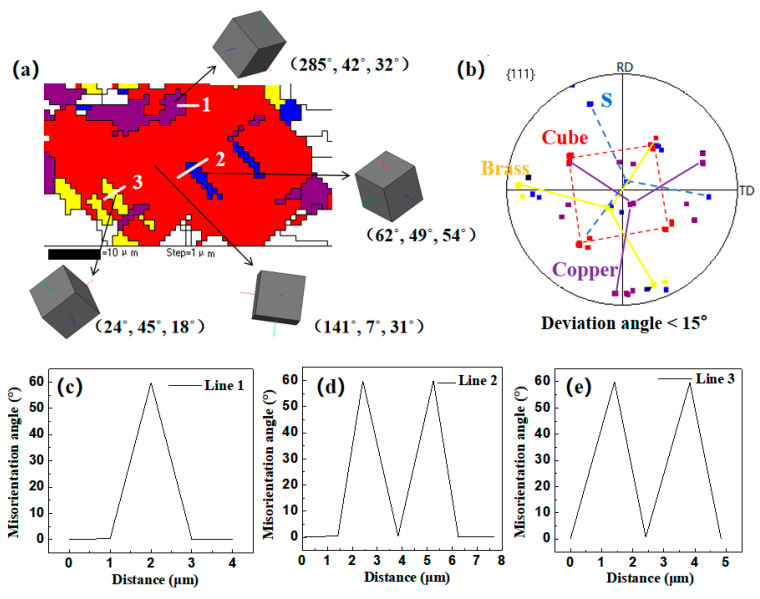
Typical region with many annealing twins selected in Figure 8d: (**a**) main texture components and their 3D crystal Cube with Euler angles; (**b**) corresponding orientations in {111} pole figure; (**c**) misorientation angles along line 1; (**d**) misorientation angles along line 2 and (**e**) misorientation angles along line 3.

Figure 12 shows a typical region with many annealing twins observed in the sample annealed at 500 °C after rolling with a reduction rate of 87%. The crystallographic orientation of the annealing twins growing within the large Cube-oriented recrystallized grain was identified as S, Copper and Brass, with confirmed misorientation angles along three white lines in Figure 12a showing a 60° <111> relationship. As the annealing temperature increased, the S-, Copper- and Brass-oriented grains were gradually engulfed by Cube-oriented grains during grain growth, leading to a gradual decrease in twin boundaries between them, as depicted in Figure 8. However, it was observed that the sample with an 87% rolling reduction still exhibited a higher density of annealing twins compared to the sample with a 60% rolling reduction at higher annealing temperatures. This suggests that the density of annealing twins after recrystallization was correlated with prior deformation levels, and more annealing twin boundaries formed in the oxygen-free copper sheets with the increase in cold-rolling reduction. 

## 4. Conclusions

1. The deformation textures of S, Copper and Brass were progressively enhanced with the increase in cold-rolling reduction. When the rolling reduction exceeded 60%, the orientations along the α-oriented fiber converged towards Brass, and the orientation density of the β fiber obviously increased.
2. The recrystallization texture was significantly influenced by the cold-rolling reduction. After a 60% cold-rolling reduction, Copper and S texture components gradually decreased, while the {011}<511> recrystallization texture component formed with an increase in annealing temperature. Following an 87% cold-rolling reduction, a strong Cube texture formed, and other textures were suppressed as annealing temperature increased. The strong Brass and S deformation texture favored the formation of a strong Cube annealing texture.
3. The annealing twin density decreased with the increase in annealing temperature, and more annealing twin boundaries formed in oxygen-free copper sheets with the increase in cold-rolling reduction.

## Figures and Tables

**Table 1:** The chemical composition of the copper sheets (wt%).

Cu	Zn	Ag	Fe	Sn	Pb	Bi	As	P	S	O
99.995	0.0005	0.0005	0.0002	0.0004	0.0004	0.0003	0.0006	0.0003	0.0006	0.0003

## Data Availability

Data are contained within the article.
